# The Impact of Perioperative Arterial Infarct on Recurrence, Functional Outcomes, and Survival in Glioblastoma Patients

**DOI:** 10.3389/fonc.2020.00706

**Published:** 2020-05-13

**Authors:** Jakob T. Lupa, Jeffrey J. Raizer, Irene B. Helenowski, Benjamin P. Liu, Kartik Kesavabhotla, Matthew C. Tate

**Affiliations:** ^1^Department of Anesthesiology and Pain Medicine, University of Washington, Seattle, WA, United States; ^2^Department of Neurology, Feinberg School of Medicine, Northwestern University, Chicago, IL, United States; ^3^Department of Preventive Medicine, Feinberg School of Medicine, Northwestern University, Chicago, IL, United States; ^4^Department of Radiology, Feinberg School of Medicine, Northwestern University, Chicago, IL, United States; ^5^Department of Radiation Oncology, Feinberg School of Medicine, Northwestern University, Chicago, IL, United States; ^6^Department of Neurological Surgery, Feinberg School of Medicine, Northwestern University, Chicago, IL, United States

**Keywords:** glioblastoma, infarct, resection, recurrence, survival

## Abstract

**Background:** Perioperative infarcts are a known complication that can occur during the resection of glioblastoma (GBM). Recent studies suggest that gross total and even “supra-total” resections may be associated with an increased survival but the rate of complications, including perioperative ischemia, may increase with these more aggressive resection strategies. However, little is known about the impact that perioperative infarcts have on survival, functional outcomes, and tumor recurrence patterns. Our study attempted to quantify and characterize the functional consequences of a perioperative infarct, as well as risk factors associated with occurrence.

**Methods:** Seventy-three patients with a diagnosis of GBM and perioperative ischemia by MRI were identified from the electronic medical record system. We obtained demographic, prognostic, and stroke risk factor data. Infarct volumes were calculated from diffusion-weighted MRI scans, and subjects were segregated into an infarct cohort or a control cohort based on whether the identified lesion appeared to be an infarct in an arterial distribution or instead appeared to be expected postoperative changes. A multivariate statistical analysis was performed on the dataset.

**Results:** Median age was 58.6 years, median post-op KPS (Karnofsky Performance Status) was 90, and median extent of resection (based on MRI) was 97.8%. Overall, perioperative arterial infarcts were uncommon (2.0%), did not have a statistically significant impact on survival (17.9 vs. 18.9 months), did not worsen neurologic function, and did not alter the pattern of recurrence.

**Conclusion:** Perioperative arterial infarcts were uncommon in our patients despite aggressive resection and when present had no impact on survival or neurologic function. Given the clear benefit of maximal tumor resection, the risk of perioperative infarct should not deter maximal safe resection.

## Introduction

Initial treatment for glioblastoma (GBM) consists of maximal safe surgical resection, which has been shown to positively impact long-term outcomes (1–11). However, one potential risk of aggressive resection is an intra- or post-operative infarct, most commonly at or near the resection site. The incidence of ischemic infarcts following glioma resection has been reported to occur in 31% of patients with a newly diagnosed glioma and 80% of patients with a recurrent tumor based on data from one center (12). Another study found that infarcts larger than 4 cm^3^ occurred in 10.2% of glioma patients who underwent surgical resection (13). Hence, infarcts can be sizable and could have a negative impact on postoperative neurological functioning and survival (particularly if they were to hasten progression or prevent patients from undergoing standard chemotherapy or radiotherapy). However, there are few data describing the clinical impact of perioperative infarcts, and so we sought to better understand how this might impact outcomes. Furthermore, recent studies suggest that survival may be increased in patients who undergo more extensive resections such as those removing tissue beyond contrast-enhancement in GBM or beyond FLAIR abnormality in low grade gliomas (14–16). For these more aggressive approaches, there is a theoretical concern of a higher complication rate relative to subtotal or gross total approaches, including the rate of perioperative ischemic stroke. It is also unclear to what extent perioperative ischemic stroke impacts a patient's subsequent radiation and chemotherapy, as the infarct zone could possibly alter regional distribution of chemotherapeutics (and perhaps therefore their efficacy) or lead to exacerbated radiation necrosis from radiotherapy. Thus, it is increasingly important that we understand the clinical implications of perioperative complications, including ischemia, as surgical resections become more extensive.

We hypothesize that patients who experience perioperative ischemic stroke might have lower overall and progression-free survival. Reasons for this effect include an upregulation of angiogenic/survival factors in the infarct zone and/or a decline in neurologic functioning. A decrease in neurologic functioning could lead to direct morbidity (such as infection, DVT/PE, lower performance status, increased hospital stay) as well as a decreased ability to undergo needed adjuvant therapy such as radiation and chemotherapy. KPS (Karnofsky Performance Status), a scale used to classify performance status, can decreased in patients who experience functional debilitation from an ischemic stroke. Because lower KPS has been shown to be associated with reduced survival in GBM patients, we hypothesize that reductions in KPS from perioperative stroke would similarly reduce survival (17). We also hypothesize that larger infarcts impose a greater likelihood of these co-morbidities. In contrast to these presumptively negative impacts of perioperative ischemia, a potential positive effect of a perioperative infarct from an oncologic perspective is destruction of occult tumor cells located in regions surrounding the primary tumor. This additional cytoreduction could thus theoretically improve survival. Additionally, we hypothesize that perioperative ischemia could decrease the rate of local recurrence due to increased tumor cell death in the area of ischemia near the resection cavity. The rate of distant recurrence would therefore increase. A recent study by Theipold et al. investigating the pattern of recurrence in GBM patients who experienced a perioperative infarct showed an increase in diffuse or distant tumor recurrence in the infarct cohort compared to controls (18). Despite this finding, perioperative infarct was not found to be associated with a change in overall survival in that study. Our study differs from the study conducted by Thiepold et al. in that we collected data on post-operative KPS and rate of new neurologic deficit to better characterize the functional impact this ischemia may have on patients in addition to the potential impact on survival. Finally, we hypothesize that patients with traditional stroke risk factors (such as a history of hypertension, hyperlipidemia, or diabetes mellitus) will have a higher rate of arterial infarct following GBM resection.

To understand the effects of surgically related infarcts, we performed a retrospective analysis of GBM patients who were found to have an infarct on post-operative MRI. In addition, our study is the first to our knowledge to clarify which patients are at highest risk for having a perioperative infarct with the aim of providing clinicians with objective information for counseling patients regarding risk of surgical resection in GBM.

## Methods and Materials

### Data Collection

This retrospective analysis was carried out in accordance with the recommendations and approval of the Northwestern University Institutional Review Board (IRB). Patient consent was not obtained given the retrospective chart review nature of the study and since the study could not be practically carried out without a waiver of consent. Using the electronic medical record system, pathology reports spanning the years 2003 to 2013 were screened for the key term “glioblastoma multiforme.” This captured subjects who had a tissue diagnosis of GBM based on the 2007 WHO classification of CNS tumors, and captured patients who may have received a diagnosis which included the older term “multiforme.” This created a set of 1,231 potential subjects who were diagnosed with a GBM. Radiology reports belonging to that subset were then screened for key terms including “infarct,” “ischemia,” and “restricted diffusion.” Flagged MRI reports were screened to exclude falsely identified reports (i.e., when “restricted diffusion” was identified because the report contained: “There is no restricted diffusion”). Seventy-six subjects were identified that fit the criteria. Among these GBM patients with verified perioperative ischemia, three subjects were excluded due to incomplete follow-up (most commonly due to the subject receiving their post-surgical oncology care at an outside location), which led to 73 subjects in our final analysis. The following data were abstracted from chart review in these patients: age at diagnosis, gender, ethnicity, progression-free survival (PFS, defined as time from diagnosis to disease progression, radiographic evidence of tumor recurrence or significant clinical deterioration), overall survival (OS), preoperative and postoperative KPS, preoperative and postoperative neurological examination discordances (to note a new deficit in the motor, sensory, or language domains), whether the infarct occurred during the patient's primary resection or a secondary resection at recurrence, if chemotherapy and radiation therapy was delayed more than 4 weeks post resection, post-operative disposition, past medical history (diabetes mellitus, hypertension, smoking history, previous thrombotic event, personal or family history of clotting disorder, family history of vascular disease, history of temozolomide/radiation, number of other medical treatments tried), tumor MGMT methylation status, and tumor IDH status.

### Tumor and Infarct Volume Calculations

The MRIs of subjects that were captured by our electronic medical record search were independently reviewed by the authors. If b1000 diffusion scans demonstrated hyperintensity surrounding the resection cavity and ADC-mapped diffusion scans demonstrated hypointensity in the same distribution, this was considered a verified infarct. The corresponding MRI reports, which were dictated by a certified neuroradiologist, were also inspected to ensure there was no disagreement in interpretation of infarct. Volume measurements on MRI (initial tumor volume, residual tumor volume after surgery, and infarct volume) were made using General Electric Healthcare's PACS software (GE Healthcare, Chicago, IL, USA). B1000 diffusion-weighted scans were used to measure infarct volume and post-contrast T1 scans were used to measure tumor volume. An area was drawn onto each axial slice of the scan to circumscribe the infarct using the polygon tool. The area of each polygon was calculated by the PACS program, and using the known slice thickness, volume of infarct or tumor was calculated using the following formula:

$$\begin{array}{l} \overset{n}{\underset{i=1}{\sum }}i=\left(\text{slice thickness}\right)*\left(\text{area of slice n}\right) \end{array}$$

To calculate extent of resection (EOR), the area of initial and residual tumor involvement was measured using the same polygon tool on axial slices of the post-contrast T1 scans performed pre-operatively and post-operatively. Initial and residual tumor volume was calculated using the same formula as was used to measure infarct. EOR was then calculated as a percentage using the following formula:

$$\begin{array}{l} \text{EOR }=\;\left(1-\frac{\text{postoperative tumor volume}}{\text{preoperative tumor volume}}\right)*100\% \end{array}$$

Lastly, pattern of recurrence was determined by comparing each subject's post-operative MRI and the MRI that ultimately demonstrated progression. The distance between the site of recurrence and the edge of the resection cavity was measured on post-contrast T1 scans. Subjects who had their recurrence within 3 cm of the resection cavity were deemed to have a local recurrence, and those with tumors beyond that threshold were deemed to have a distant recurrence. This 3 cm threshold is higher than the 1 cm threshold used by Thiepold et al. because this would provide for higher specificity in the analysis of the rate of distant recurrence than a 1 cm threshold would (17).

### Cohort Segregation

**Figure 1:**
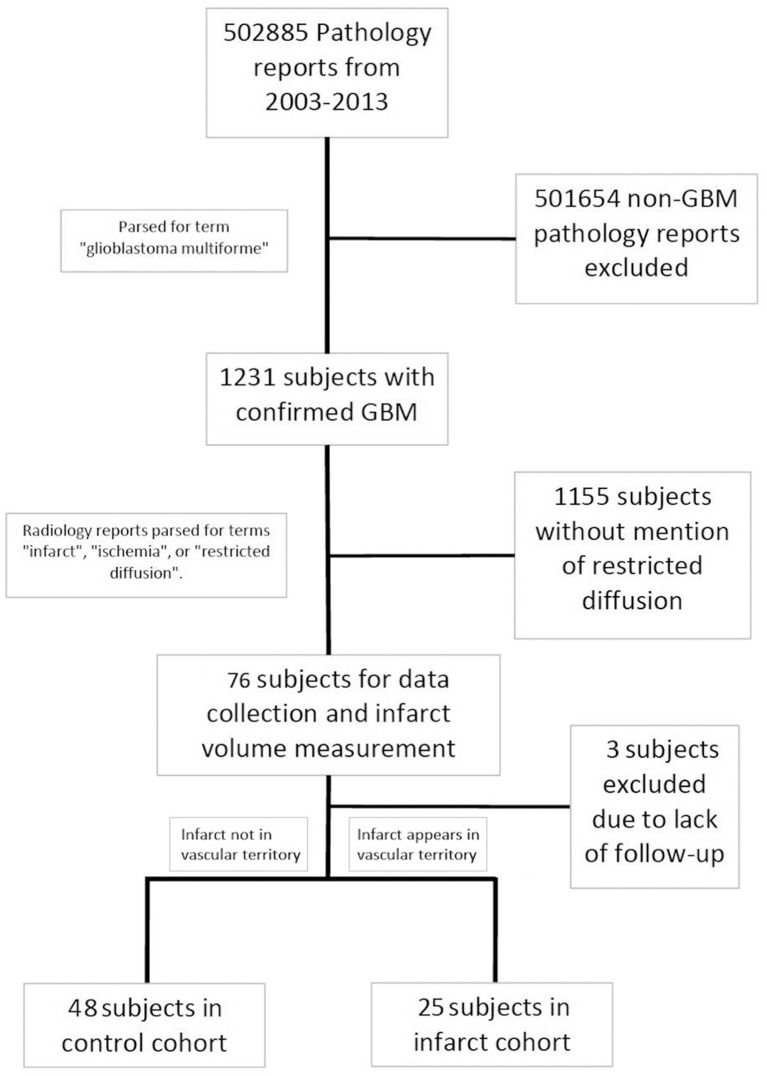
Process used to build study cohorts.

**Figure 2:**
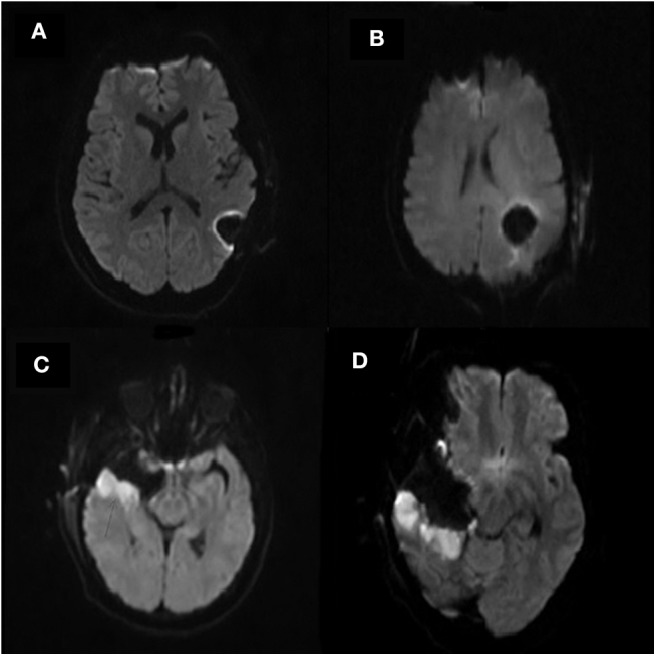
**(A–D)** Selected example post-operative diffusion (b1000) MRI scans of four separate subjects. **(A,B)** are representative of subjects in the control cohort. Note the resection cavities seen in the left parietal lobe on both scans which exhibit a minimal rim of hyperintensity surrounding the resection cavity. **(C,D)** are representative of subjects in the infarct cohort. Note the resection cavity seen in the right temporal lobe on both scans. Also note the larger area of diffusion hyperintensity surrounding the resection cavity representing arterial infarcts.

Subjects were segregated into either an infarct cohort or a control cohort based on the radiographic pattern of their infarct (Figure 1). Subjects were placed into the infarct cohort if the shape of the infarct appeared to be wedge-shaped such that it could occur from occlusion of a large or medium source vessel feeding a vascular territory (*n* = 25). The rest of the subjects that had expected post-operative changes, including those with a thin rim of restricted diffusion around the resection cavity, were placed into the control cohort (*n* = 48). We have provided two examples of the typical imaging pattern consistent with these subjects in the control cohort and contrasted them with two examples of the typical imaging pattern seen in subjects placed in the infarct cohort (Figures 2A–D).

Both a univariate analysis and a multivariate statistical analysis were performed to establish the impact of perioperative ischemic stroke on survival, neurologic outcomes, and recurrence patterns, as well as to identify risk factors for developing a post-operative infarct.

### Statistics

All statistical analyses were performed by a biostatistician (I.H.). In our univariate analysis, the Wilcoxon rank sum test was used for continuous variables and Fisher's exact test was used for binary and categorical variables. The log-rank test was used in the analysis of our Kaplan-Meier curves in a univariate setting. Lastly, Cox regression models were used in the multivariate analysis of our data. Our prospectively determined *p*-value for significance was *p* = 0.05. Analyses were conducted in SAS 9.4 (SAS Institute Inc., Cary, NC, USA).

## Results

### Preoperative Factors Predicting Perioperative Stroke

A total of 73 subjects were included in the analysis. Median age for the study population was 58.6 years, median pre-op KPS was 80 and median post-op KPS was 90. All patients had a diagnosis of GBM confirmed by a certified neuropathologist. Preoperative characteristics for the control and infarct cohorts and univariate analysis of differences between groups are reported in tabular format (Table 1). There were no significant differences in age, gender, pre-operative KPS, or pre-operative tumor volume between the infarct and control groups. Subjects in the infarct cohort were less likely to have a personal history of hyperlipidemia than those in the control cohort (16.0 vs. 41.7%, *p* = 0.04). Finally, we found a significant increase in the proportion of subjects of non-Caucasian race in the infarct group (*p* = 0.04).

**Table 1:** Cohort characteristics.

**Variable**	**Control cohort, *n* = 48**	**Infarct cohort, *n* = 25**	***p*-value**
Age at diagnosis, median (IQR)	59 (51, 69)	55 (50, 64)	0.38
Tumor volume at resection (cm^3^), median (IQR)	35.5 (20.3, 57.4)	33.1 (15.1, 63.1)	0.93
Ethnicity			**0.04**
Caucasian	40 (83.3%)	16 (64.0%)	
Non-caucasian	2 (4.2%)	6 (24.0%)	
Declined	6 (12.5%)	3 (12.0%)	
Gender			0.43
F	14 (29.2%)	10 (40.0%)	
M	34 (70.9%)	15 (60.0%)	
Pre-op KPS, median	80	90	0.18
Infarct occurred during primary resection	45 (93.8%)	22 (88%)	0.33
Standard therapy received post-surgery (Temozolomide + Radiotherapy)	44 (91.7%)	24 (96.0%)	0.65
Intra-operative mapping performed	4 (8.3%)	0 (0%)	0.50
Tumor MGMT methylation positive	7 (14.6%)	3 (11.1%)	0.86
Tumor IDH mutation positive	2 (4.2%)	0 (0%)	0.62
Residual tumor volume post resection (cm^3^), median (IQR)	0.5 (0, 2.4)	0.8 (0.8, 2.7)	0.45
Extent of resection (%), median (IQR)	98.1 (89.9, 100)	96.9 (90.5, 99.6)	0.93

Measurable volume of infarct on MRI ranged from 1.9 to 49.2 cm^3^ (median 12.2 cm^3^). Median extent of resection did not differ significantly between cohorts (98.1% in control cohort vs. 96.9% in infarct cohort, *p* = 0.93). There were also no differences between control and infarct groups with respect to personal history of ischemic stroke risk factors (except for hyperlipidemia) or family history of vascular/hematologic disorders (Table 2). Lastly, there were no differences between infarct and control groups in tumor MGMT methylation status (11.1 vs. 14.6% methylated, *p* = 0.86), IDH mutation status (0 vs. 4.2% positive, *p* = 0.62), or proportion of subjects who underwent chemotherapy and/or radiotherapy.

**Table 2:** Stroke risk factors.

**Variable**	**Control cohort, *n* = 48**	**Infarct cohort, *n* = 25**	***p*-value**
Personal history of:			
Diabetes mellitus	5 (10.4%)	2 (8.0%)	0.99
Hypertension	20 (41.7%)	8 (32%)	0.46
Coronary artery disease	4 (8.3%)	0 (0%)	0.29
Hyperlipidemia	20 (41.7%)	4 (16.0%)	**0.04**
Smoker (ever)	16 (33.3%)	10 (40.0%)	0.75
Previous thrombotic event	12 (25.0%)	5 (20.0%)	0.85
Clotting disorder	0 (0%)	1 (4.0%)	0.34
Family history of vascular disease	5 (10.4%)	6 (24.0%)	0.17

### Overall Survival, Progression-Free Survival, and Functional Outcome

**Figure 3:**
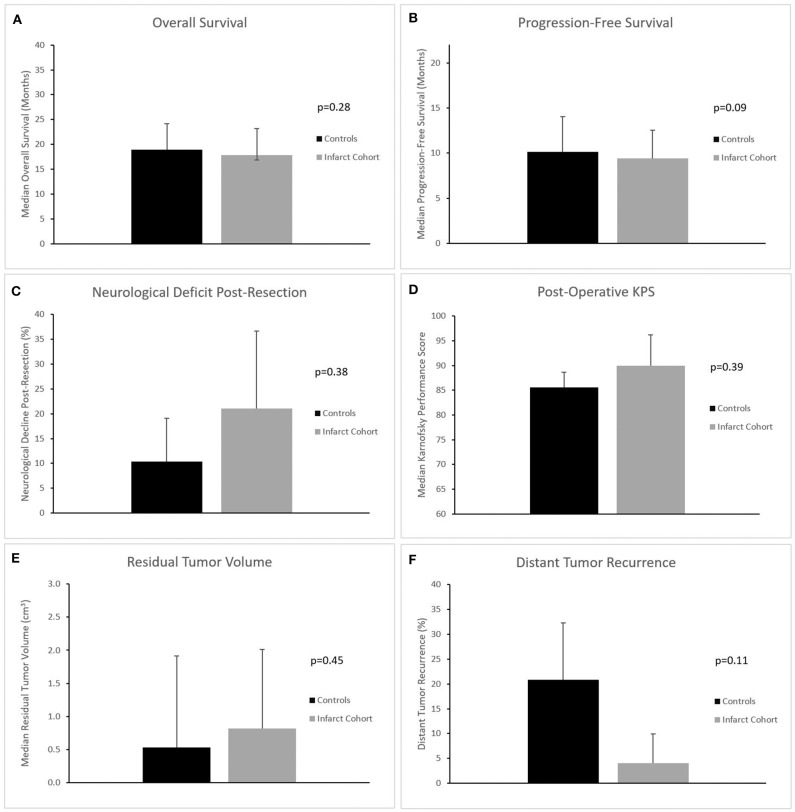
**(A–F)** Bar graphs comparing overall survival, progression-free survival, rate of neurological deficit, postoperative KPS, residual tumor volume post-resection, and rate of distant tumor recurrence between cohorts. There were no significant differences noted between the two cohorts in any of these variables.

We assessed postoperative outcomes using univariate analysis. Median overall survival was not different between infarct and control groups (17.9 vs. 18.9 months, *p* = 0.28), (Figure 3A). Similarly, median progression-free survival was not different between infarct and control groups (9.4 vs. 10.1 months, *p* = 0.09), (Table 3 and Figure 3B).

**Table 3:** Survival outcomes.

**Variable**	**Control cohort, *n* = 48**	**Infarct cohort, *n* = 25**	***p*-value**
Overall survival (months)			0.28
Mean & S.E.	21.5 (2.7)	17.8 (2.7)	
Median & 95% CI	18.9 (15.5, 21.9)	17.9 (14.3, 20.6)	
Progression-free survival (months)			0.09
Mean & S.E.	11.6 (2.0)	7.8 (1.6)	
Median & 95% CI	10.1 (8.3, 13.6)	9.4 (4.8, 15.0)	

**Figure 4:**
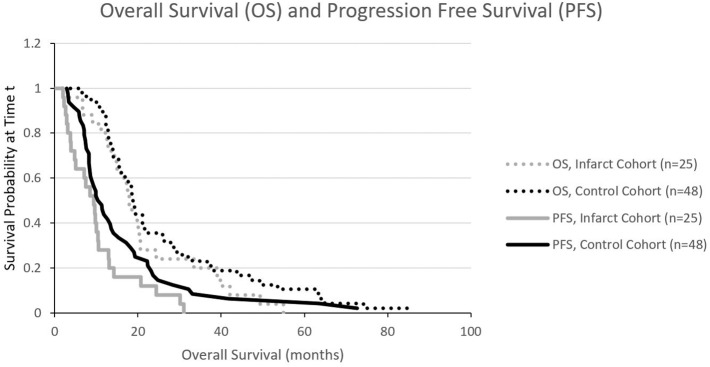
Kaplan Meier curves illustrating progression free survival (PFS) and overall survival (OS). There is no significant difference in PFS or OS.

We also constructed Kaplan Meier survival curves to investigate for differences in both overall survival and progression free survival between cohorts at various time points following resection (Figure 4). There were no statistically significant differences in median overall survival (*p* = 0.28) or median progression-free survival (*p* = 0.09) between infarct and control cohorts at the 1, 2, and 5-year time points.

In our multivariate analysis, the only variable found to impact overall survival was non-Caucasian race (HR = 1.885, CI = 1.021–3.479, *p* = 0.04). No variables were found to impact progression-free survival in our multivariate analysis.

With regard to functional outcomes, there were no differences observed when comparing rate of post-op neurologic deficit (*p* = 0.38), post-op KPS (*p* = 0.39), or post-discharge disposition (*p* = 0.96), (Table 4 and Figures 3C,D). There were also no differences between proportion of subjects receiving standard oncologic therapy (temozolomide + radiation therapy post-resection), (*p* = 0.65), extent of resection (*p* = 0.93), or residual tumor volume (*p* = 0.45), (Figure 3E).

**Table 4:** Outcome measures with univariate analysis.

**Variable**	**Control cohort, *n* = 48**	**Infarct cohort, *n* = 25**	***p*-value**
Infarct volume (cm^3^), median (IQR)	-	12.2 (8, 21.2)	-
Post-op KPS, median (range)	90 (60, 100)	90 (20, 100)	0.39
Neurologic deficit post-resection	5 (10.4%)	5 (20.0%)	0.38
Motor deficit	4 (8.3%)	4 (16%)	0.23
Sensory deficit	0 (0%)	0 (0%)	0.26
Language deficit	1 (2.1%)	1 (3.7%)	0.25
Post-operative disposition			0.96
Home	38 (79.2%)	23 (85.2%)	
Acute inpatient rehabilitation	6 (12.5%)	3 (11.1%)	
Subacute nursing facility	1 (2.1%)	0 (0%)	
Other	3 (6.3%)	1 (3.7%)	
Standard therapy received post-surgery (Temozolomide + Radiotherapy)	44 (91.7%)	24 (96.0%)	0.65
Delay to radiation therapy beyond 4 weeks	13 (27.1%)	5 (20%)	0.67
Pattern of recurrence			0.11
Local	37 (77.1%)	24 (96.0%)	
Distant	10 (20.8%)	1 (4.0%)	

### Recurrence Pattern

Finally, we also sought to determine the effect of perioperative infarcts on tumor recurrence patterns. Univariate analysis showed no significant difference in rate of local recurrence between cohorts (as determined by tumor recurrence within 3 cm of the initial resection cavity), (*p* = 0.11), (Figure 3F).

## Discussion

In the current series, 6.2% of GBM patients in our 10-year span had perioperative infarcts identified by neuroradiologists on post-operative imaging, although most of these infarcts were small, with only 3.5% of patients having an infarct volume >3 cm^3^. We focused our study on the patients who had a perioperative infarct that specifically appeared in an arterial pattern for two reasons. First, we hypothesized that those patients were the most likely to experience a bad outcome from their infarct given that these arterial infarcts tended to be larger than infarcts that manifested as a small rim of restricted diffusion around the resection cavity. Secondly, we hypothesized that there may be a common mechanism for the occurrence of these vascular infarcts and thus perhaps we could identify patient risk factors that may predict which patients were more likely to experience them.

These perioperative infarcts had no negative effect on overall survival or progression-free survival, which may be due to the fact that there were no true large vessel (such as MCA) infarcts. This is consistent with recent data by Thiepold et al. that did not find a statistically significant difference in overall survival in patients who experienced a perioperative infarct (18). In addition, having a perioperative infarct did not deter patients from receiving standard of care or adjuvant treatment, nor did it worsen postoperative neurologic function or alter the disposition of patients after their surgery. Finally, it should be noted that the lack of clinical impact of perioperative ischemic strokes was not the result of confounding due to a less aggressive resection strategy, as the median extent of resection for the infarct and control cohorts were 96.9 and 98.1%, respectively. Thus, aggressive surgical resection using modern techniques (e.g., neuronavigation, functional mapping) results in acceptable morbidity profiles related to vascular events.

Given the clear relationship of extent of tumor removal and survival as well as recent studies demonstrating improved survival with “supra-total” resections (defined as resection of surrounding FLAIR+, non-enhancing tumor in addition to the typical contrast-enhancing surgical target), there is a trend toward more aggressive resections in patients with GBM (14–16). It is possible that the portion of patients experiencing perioperative infarct may increase with supratotal resection or aggressive resection. However, in our subjects, extent of resection did not predict infarct occurrence and if an infarct did occur there was generally no apparent clinical consequence to the patient. Of course, if a small infarct were to occur in a critical brain region, then we would expect to see a clinical impact. This, however, was not apparent in our study cohort. In other words, given a clear benefit of aggressive surgery and minimal risk of perioperative infarct occurrence/impact, aggressive surgical resection with or without the aim of supra-total resection is reasonable.

We sought to define risk factors that might predict a patient's likelihood to experience a perioperative infarct since this could aid in counseling patients regarding risks of surgery. Risk factors that we hypothesized would be most likely to correlate with perioperative infarct were larger tumor size, advancing age, and personal history of various stroke risk factors (such as personal history of hypertension or diabetes). Resections of larger tumors have a higher probability of approaching critical vascular structures and thus we expected to find a larger average tumor volume in our infarct cohort. Also, advancing age is a well-known risk-factor for cardiovascular disease (CVD), and is one of the factors used in the Framingham Risk Score to predict future CVD (including ischemic stroke) (19). Thus, we expected advancing age to be a risk factor for perioperative infarct. However, none of these risk factors were found to be more prevalent in the infarct cohort. These findings suggest that it may not be possible for clinicians to predict which patients might experience perioperative infarct based on the large number of risk factors we considered in our retrospective cohort study.

Another goal of our study was to identify a possible mechanism for these perioperative infarcts. It has been shown that those of a non-Caucasian race (specifically Hispanic or African-American race) are at a significantly elevated risk for stroke originating from an intracranial atherosclerotic plaque, and non-Caucasian race is the only risk factor for infarction that we identified in the analysis for our study population (20). However, mechanical disruption of atherosclerotic plaques in larger arteries (such as the ACA and MCA) would be unlikely to cause the peri-cavity pattern of infarct seen in those who underwent GBM resection (see Figures 2C,D). Disruption of fibroid angiopathy occurring in smaller penetrating arteries (such as that which causes lacunar strokes) is another potential mechanism for these infarcts, though it is unknown whether or not these fibrinoid lesions would be susceptible to mechanical disruption during surgery. Lastly, vasospasm remains a potential etiology of post-resection ischemia. However, data from studies investigating subarachnoid hemorrhage demonstrate elevated risk of vasospasm in younger patients, and our study did not show age (old or young) to influence the incidence of perioperative infarct (21). While it is difficult to identify the source of perioperative infarct in patients who underwent a surgical resection for GBM, vasospasm, mechanical disruption of intracranial plaques or trauma to perforators all remain possible etiologies.

Given recent data from Thiepold et al. showing that GBM patients who experienced perioperative ischemia were more likely to have a distant or diffuse recurrence than those without perioperative ischemia, we examined the effect of infarct on tumor recurrence pattern in our series (18). Our data did not show a significant change in the proportion of local recurrence between control and infarct groups, suggesting that perioperative infarct does not impact recurrence site. However, it is possible that this discrepancy in results is due to a difference in definition of tumor recurrence location. Aside from “local” and “distant” recurrences, Thiepold et al. defined a third category of recurrence titled “diffuse” which was grouped with “distant” recurrences during analysis to be compared with patients who had a local recurrence. Their combined “diffuse or distant” definition was not exclusive of recurrences located within 3 cm of the primary site (as our “distant” definition was), and thus this difference in definition may be responsible for the discordant results. In summary, our data shows that local ischemia does not result in recurrence sites that are more distant from the primary site. Rather, recurrences are most commonly local as is typical for GBM.

Finally, in addition to survival outcomes, we investigated the neurologic consequences imparted by perioperative infarcts. We did not observe a significant difference between cohorts for postoperative KPS, rate of new neurologic deficits, or eventual post-discharge disposition. This suggests that perioperative strokes do not dramatically alter functional outcomes. In addition, only one of our 25 subjects in the infarct cohort had a KPS <60 at least 2 weeks out from discharge (compared to none in the control cohort) and so the occurrence of highly debilitating infarcts appears to be rare.

There were several limitations to our study. First, we were only able to identify a small number of patients who experienced a significant infarct and therefore our statistical power was limited. In addition, our control group consisted of subjects who initially screened into our study based on our parsing of radiology reports but subsequently were found to not have an arterial infarct based on manual review of their post-operative imaging, resulting in at least some degree of selection bias. Furthermore, not every patient with a rim of diffusion restriction around their cavity may have had that commented on in their radiology report given that it can be an expected post-surgical finding and an inter-reviewer variability exists between neuroradiologists in their habits of commenting on specific expected findings. This could have led to a bias in patient selection as perhaps there were other patients with these expected findings who were not included in the control group because their radiology reports were not flagged during our initial parsing. Another limitation of our study is that the majority of our patients had excellent neurologic function prior to surgery, and thus our data may lack relevance for patients with significant deficits prior to surgery that presumably had tumor invading functional areas and may have been at higher risk for ischemia-induced functional decline. It is also possible that patients with very small infarcts (which would likely be captured in our control group) could nonetheless have significant functional decline (or even a reduction in overall survival) if that infarct were in certain critical brain regions, such as the internal capsule, and such effects would not be captured by our analysis. Our study also did not correlate infarct location with severity of functional debilitation, though a larger sample size would be needed to study this potential effect given that only three of our 73 subjects experienced a severely debilitating stroke. Lastly, while our study did not show a statistically significant difference in OS and PFS between cohorts, there did appear to be a potential trend toward significance between these two variables. This may warrant further study of the subject with a larger sample size to more definitively determine the impact of this perioperative finding. Such a study would likely require a collaborative effort between institutions to generate a larger subject pool given that a comparable study from a large academic institution in Germany had a similar sample size to ours (*n* = 92 vs. *n* = 73) (17). A multi-institutional study with a significantly larger sample size could also study the potential correlation between infarct location and significant functional decline.

In summary, our study has shed some light on an infrequent and not well-reported phenomenon in GBM patients who undergo aggressive surgical resection. Arterial infarcts are uncommon (2.0% in our overall GBM population) and were shown to generally have no negative impact on overall survival and functional outcome, even despite aggressive surgical resection in the 73 subjects that were included in the final analysis (overall median EOR = 97.8%). The only risk factor identified in our study that positively predicted perioperative infarct was non-Caucasian race. This suggests that it does not appear to be possible to use traditional stroke risk factors to identify patients who are at a meaningfully higher risk of perioperative infarct. Thus, perioperative infarct should remain in the preoperative discussion of risks and benefits for all patients who are preparing to undergo resection of a GBM. It should be reassuring to patients that these infarcts, while rare, have not been shown to significantly alter survival or functional outcome.

## Data Availability Statement

The datasets analyzed for this study are unavailable for sharing due to privacy, ethical, and legal restrictions. Requests for the data can be made to the corresponding author.

## Ethics Statement

The studies involving human participants were reviewed and approved by Northwestern University Institutional Review Board (IRB). Written informed consent for participation was not required for this study in accordance with the national legislation and the institutional requirements.

## Author Contributions

JL, JR, BL, KK, and MT contributed to the conception and design of the study. JL and KK organized the database. IH performed the statistical analysis. JL wrote the first draft of the manuscript. JR, MT, and IH wrote sections of the manuscript. All authors contributed to manuscript revision, read and approved the submitted version.

## Conflict of Interest

The authors declare that the research was conducted in the absence of any commercial or financial relationships that could be construed as a potential conflict of interest.
